# Case of Poisoning by Arsenic, Successfully Treated with Freshly Prepared Hydrated-per-oxide of Iron

**Published:** 1847-04

**Authors:** C. A. Hall

**Affiliations:** Northampton, Mass.


ART. IV.—*Case of Poisoning by Arsenic, successfully treated with freshly prepared Hydrated-per-oxide of Iron.* By C. A. HALL, M. D.

White oxide of Arsenic had been obtained for destroying rats. The label and the paper containing it had been partially destroyed by the corrosive action of the Arsenic, and it had been poured out into a tea cup, and put aside to be re-labeled at some future time. Subsequently, the family removed to another house, and in the confusion arising therefrom, the Arsenic was placed near a teacup of the same color containing super carbonate of Soda.

On the 25th of December, 1846, half a teaspoonfull of the Arsenic, probably about 80 grains, mistaking it for the sup. carbonate of Soda, was put into a pudding, the greater part of which was afterwards eaten by two persons. In less than half an hour, severe and distressing sickness came on, with vomiting and great prostration. Being alone in the house, and unable even to raise a window to call assistance, they remained for some time in this dangerous situation. Fortunately, a sister who had been absent, returned home, and in a few minutes afterwards I was at the house. Finding that Arsenic had been taken in so large a quantity, and knowing of but one perfectly effectual antidote, I at once proceeded to prepare a quantity of the hydrated peroxide of Iron, which I administered with an unsparing hand. In less than half an hour decided relief was obtained, the retching and vomiting though still kept up, occurred at longer intervals, and with less severity. The pain and faintness continued more or less during the night, yet the relief already apparent, induced me to continue the use of the antidote. The next day the vomiting occurred but once or twice, and in one only of the persons. In the mean time, however, a new train of symptoms came on. The tongue was swollen, there was a burning pain and considerable inflammation in the throat, accompanied with great thirst, and in one of the cases with hiccough, and much tenderness of the Epigastrium. The evacuations from the bowels were dark and offensive, and attended with pain and tenesmus.—There was for a long time great prostration of strength; indeed, neither of the persons have felt quite well until within a few days.

I am induced to make these cases public, not only to give additional evidence of the efficacy of the *freshly prepared* hydrated peroxide of Iron, as an antidote to poisoning by white oxide of Arsenic; but, also, since it is well known that the remedy is of little use except when *freshly prepared*, to urge it upon every Medical man, not only to bear in mind how it is prepared, but actually to go through the manipulations, and prepare it, once at least, for himself. Then, when it is wanted, as it always is in haste, and when there is no time to look for specific directions, he can prepare it more dextrously, and with no doubts or misgivings as to its purity.

Lest there may be some one of your readers who has not a formula for preparing the Hydrated peroxide of Iron, I subjoin that of the last edition (1845) of the U. S. Pharmacopœia. It has been well suggested that the articles for making it should be kept in vials, in the proper proportions, ready for use at a moment's notice:

"℞ Sulphate of Iron, ℥ iv.

Sulphuric Acid, f ʒ iiiss.

Nitric Acid, f ʒ vi. or q. s.

Solution of Ammonia, q. s.

Water, Oii.

Dissolve the Sulphate of Iron in the water, add the Sulphuric Acid, and boil the solution; then add the Nitric Acid in small portions, boiling the liquid for a minute after each addition, until the acid ceases to produce a dark color. Filter the liquid, allow it to cool, and add solution of Ammonia in excess, stirring the mixture briskly; wash the precipitate with water until the washings yield no precipitate with Chloride of Barium."

In the foregoing case I used no Sulphuric Acid. I did not filter the liquid, or wait for it to cool, nor did I stop to test it with Chloride of Barium ; but washed it three or four times with water, poured it on cotton cloth, and administered it while hot. Doubtless the better plan would be to follow exactly, the directions of the Pharmacopœia.

NORTHAMPTON, MASS., *March* 18*th*. 1847.

